# Abnormal Topology of the Structural Connectome in the Limbic Cortico-Basal-Ganglia Circuit and Default-Mode Network Among Primary Insomnia Patients

**DOI:** 10.3389/fnins.2018.00860

**Published:** 2018-11-23

**Authors:** Yunfan Wu, Mengchen Liu, Shaoqing Zeng, Xiaofen Ma, Jianhao Yan, Chulan Lin, Guang Xu, Guomin Li, Yi Yin, Shishun Fu, Kelei Hua, Chao Li, Tianyue Wang, Cheng Li, Guihua Jiang

**Affiliations:** ^1^The Second School of Clinical Medicine, Southern Medical University, Guangzhou, China; ^2^Department of Medical Imaging, Guangdong Second Provincial General Hospital, Guangzhou, China

**Keywords:** primary insomnia, diffusion tensor imaging, human connectome, fMRI, limbic system

## Abstract

**Purpose:** Primary insomnia (PI) is the second most common mental disorder. However, the topologic alterations in structural brain connectome in patients with PI remain largely unknown.

**Methods:** A total of 44 PI patients and 46 age-, gender-, and education level matched healthy control (HC) participants were recruited in this study. Diffusion tensor imaging (DTI) and resting state MRI were used to construct structural connectome for each participant, and the network parameters were employed by non-parametric permutations to evaluate the significant differences between the two groups. Relationships between abnormal network metrics and clinical characteristics, including the disease duration, the Pittsburgh Sleep Quality Index (PSQI), the Insomnia Severity Index (ISI), the Self-Rating Anxiety Scale (SAS), and the Self-Rating Depression Scale (SDS), were investigated with Spearman’s correlation analysis in PI patients.

**Results:** PI patients demonstrated small-world architecture with lower global (*P* = 0.005) and local (*P* = 0.035) efficiencies compared with the HC group. The unique hub nodal properties in PI patients were mainly in the right limbic cortico-basal-ganglia circuit. Five disrupted subnetworks in PI patients were observed in the limbic cortico-basal-ganglia circuit and left default-mode networks (DMN) (*P* < 0.05, NBS corrected). Moreover, most unique hub nodal properties in the right limbic cortico-basal-ganglia circuit were significantly correlated with disease duration, and clinical characteristics (SAS, SDS, ISI scores) in PI processing.

**Conclusion:** These findings suggested the abnormal anatomical network architecture may be closely linked to clinical characteristics in PI. The study provided novel insights into the neural substrates underlying symptoms and neurophysiologic mechanisms of PI.

## Introduction

Primary insomnia is the second most common mental disorder (Wittchen et al., 2011). PI is characterized by difficulty in initiating and maintaining sleep and early morning awakening for at least 3 months (American Psychiatric Association, 2013). Insomnia profoundly affects health and aging (Musiek and Holtzman, 2016) and is a significant risk factor for the development of other medical or psychiatric diseases. Although previous studies (Riemann et al., 2009, 2010, 2015; Levenson et al., 2015) have reported that heritability, polygenic vulnerability, specific cellular mechanisms and hyperarousal are involved in the pathophysiology of insomnia, the underlying neural substrate symptoms and pathological mechanisms of PI are not fully elucidated (Levenson et al., 2015; Morin et al., 2015).

Rapid development of neuroimaging technologies has provided diverse tools to non-invasively assess abnormal brain activity and anomalous structure for exploring the pathophysiology of insomnia. Two positron emission tomography studies (Nofzinger et al., 2004, 2006) have demonstrated that compared with healthy control participants (HCs), 18-fluorodeoxyglucose metabolism was lower in the prefrontal cortex while awake, and higher in the thalamus (THA), anterior cingulate gyrus, temporal region and pontine tegmentum during non-rapid eye movement sleep in PI patients (PIs). Wang T. et al. (2016) found that regional homogeneity values were higher in the left insula (INS.L), right anterior cingulate gyrus (ACG.R), left insula, bilateral precentral gyrus and left cuneus (CUN.L) in PIs than in HCs. They further used seed-based functional connectivity (FC) analysis and reported that the increased FC between left insular connectivity with many brain regions was related to emotional scores in PIs, primarily in the bilateral anterior cingulate cortex, bilateral thalamus, right fusiform, and middle temporal gyrus (Wang et al., 2017). A surface-based approach reported (Joo et al., 2014) that hippocampal volume decreased in PIs compared with that in HCs. However, a voxel-based morphometric (VBM) study (Li M. et al., 2018) found that gray matter volumes increased in the hippocampus (HIP) and decreased in the dorsolateral prefrontal and middle cingulate cortices compared with the HC group. These results were inconsistent and were mainly focused on single or few seed regions. Recently, many researchers have supported the presence of complex and advanced neural networks in different human brain regions. Brain hub regions particularly play important roles in information integration and are susceptible to attack during disease progression (Crossley et al., 2014). Thus, we speculated that brain network analysis may be more suitable to elucidate the pathophysiological mechanisms of PI.

Diffusion tensor imaging tractography can identify changes in the WM microstructure, cerebral anatomical connections and neural circuits *in vivo*. Using DTI, Spiegelhalder et al. (2014) demonstrated reduced integrity of WM tracts in the anterior internal capsule, indicating that disturbed frontosubcortical connectivity is a cause or consequence of PI. Similarly, Li et al. (2016) found that the integrity of the right lateralized WM was disrupted in PIs on DTI and tract-based spatial statistics, which involved the right internal capsule, right corona radiate, right superior longitudinal fasciculus, corpus callosum body and right thalamus (THA.R). These two studies demonstrated that DTI could detect changes and provide objective evidence to enhance our understanding of the underlying neurobiological mechanisms of PI. However, these studies focused on the reduced integrity of WM tracts, especially with regard to the internal capsule, and the results revealed no interconnections between abnormal WM tracts and cortical regions. Currently, anatomical networks constructed using DTI tractography successfully identify neuropathological changes in diverse diseases, such as Alzheimer’s disease (AD) (Shu et al., 2018), schizophrenia (van den Heuvel et al., 2013), post-traumatic stress disorder (Suo et al., 2017), and other such diseases. Lu et al. (2017a) already demonstrated disrupted structural connectivity in healthy adults with insomnia. However, topological alterations in the anatomical network, including the hub regions, remain largely unknown in PIs.

Considering these findings, we hypothesized that the PI-related brain WM network exhibits some abnormal network properties, especially in the hub regions, which are more closely related to the severity of insomnia or emotional disorders. We thus aimed to investigate the anatomical brain networks in PI patients, using DTI and graph theory analysis.

## Materials and Methods

### Subjects

At the beginning, this prospective study recruited 100 right-handed individuals between April 2010 and April 2016. Adequate images of subjects could not be obtained because of severely head movement or brain lesions as detected by MRI. In the final analyses, the study included 44 PI patients and 46 age-, gender-, and education level matched subjects. This study was approved by the Ethics Committee of the Guangdong Second Provincial General Hospital. All participants signed informed consent forms to participate in this study.

The diagnosis of PI was performed by two neurologists with 15 years of experience. PI patients were enrolled in this study according to the following criteria: (a) patients were diagnosed and confirmed as PI based on the criteria of Diagnostic and Statistical Manual of Mental Disorders, version 5 (DSM-V) (American Psychiatric Association, 2013); (b) patients with a self-complaint of difficulty falling asleep, maintaining sleep or early awakening for at least 3 months; (c) patients with no other sleep disorders, such as hypersomnia, parasomnia, obstructive sleep apnea, or sleep-related movement disorder; (d) patients with no serious organic diseases or no severe mental diseases, such as brain stroke, depression (SDS < 70), and anxiety (SAS < 70); (e) all participants were right-handed according to the Edinburgh handedness inventory; (f) all subjects were aged 18–60 years. And HC subjects have good sleep quality and the Insomnia Severity Index (ISI) score < 7 or the Pittsburgh Sleep Quality Index (PSQI) score < 7, who were enrolled from the local community.

All participants were excluded according to the following criteria: (a) pregnant, nursing or menstruating females; (b) subjects who had an abnormal signal as verified by conventional T1- or T2-weighted fluid-attenuated inversion recovery MR imaging; (c) patients who had severe brain lesions as detected by MR; and (d) the subjects who had head motion of more than 1.5 mm or 1.5° during MR imaging.

### Assessment of Sleep Situation and Mental Status

Each participant was asked to complete the PSQI, ISI, SAS, and SDS to determine the scores for estimating sleep quality and mental status prior to MRI.

### MR Data Acquisition

MR images of all PIs and healthy control participants (HCs) were obtained on a Philips 1.5T MRI system (Achieva Nova-Dual; Best, Netherlands) at the Department of Medical Imaging, Guangdong Second Provincial General Hospital. Each participant was placed in the supine position with eyes closed and the head snugly restricted by a belt and foam pads. T1WI were acquired with the following parameters: TR, 25 ms; TE, 4 ms; matrix, 256 × 256; FOV of 230 mm × 230 mm; a flip angle of 30°; section thickness, 1 mm; 160 transverse sections without gap covering the whole brain. DTI images were collected using an echo planar imaging sequence with the following parameters: TR, 10,700 ms; TE, 80 ms; FOV of 256 mm × 256 mm; matrix size of 128 × 128; a flip angle of 90°; section thickness, 2 mm; b-value, 1000 s/mm^2^ together with an acquisition without diffusion weighting (b-value = 0); 75 transverse sections without gap covering the whole cerebellum. All images were reviewed and verified by two radiologists with more than 10 years of experience.

### Data Preprocessing and DTI Network Construction

According to Cui et al. (2013), we preprocessed and analyzed all raw DTI and T1 data with the Diffusion Toolkit 0.6.4 in PANDA software (Cui et al., 2013). Because images of the cerebellum were incomplete, a WM deterministic fiber tracking approach using by PANDA software was used to construct a weighted network including 90 nodes without the cerebellum for each subject, which were defined by the Automated Anatomic Labeling (AAL90) template (Tzourio-Mazoyer et al., 2002). The abbreviations and full names of the AAL90 template are listed in Table 1. It was terminated if the WM deterministic fiber tracking with a turned angle greater than 45° or a voxel with a FA less than 0.2 by Continuous Tracking (FACT) algorithm (Chen et al., 2013). In lined with several previous brain DTI network studies (Lo et al., 2010; Wang X. N. et al., 2016; Lu et al., 2017a), we defined the weight of each effective edge between two nodes (i and j) of the WM structure as the product of FA and the fiber number (FN) along the fiber bundles, and normalized by the average volume of the two connecting regions (wi j = FN^∗^ FA/volume). As previous studies (Chen et al., 2013; Wang X. N. et al., 2016), the threshold value for the FN between two regions was defined as 3. Therefore, a weighted matrix of 90 × 90 WM structural network was constructed for each subject. After PANDA generated registration images for quality inspection, these images of each subject were carefully checked to ensure registration and segmentation quality by a radiologist with 15 years of experience.

**Table 1:** All cortical and subcortical regions with abbreviations and full name in the AAL-90 templates defined in our study.

Index	Regions abbreviations	Brain regions (full name)
1	PreCG	Precentral gyrus
2	SFGdor	Superior frontal gyrus, dorsolateral
3	ORBsup	Superior frontal gyrus, orbital part
4	MFG	Middle frontal gyrus
5	ORBmid	Middle frontal gyrus, orbital part
6	IFGoperc	Inferior frontal gyrus, opercular part
7	IFGtriang	Inferior frontal gyrus, triangular part
8	ORBinf	Inferior frontal gyrus, orbital part
9	ROL	Rolandic operculum
10	SMA	Supplementary motor area
11	OLF	Olfactory cortex
12	SFGmed	Superior frontal gyrus, medial
13	ORBsupmed	Superior frontal gyrus, medial orbital
14	REC	Gyrus rectus
15	INS	Insula
16	ACG	Anterior cingulate and paracingulate gyrus
17	DCG	Median cingulate and paracingulate gyrus
18	PCG	Posterior cingulate gyrus
19	HIP	Hippocampus
20	PHG	Parahippocampal gyrus
21	AMYG	Amygdala
22	CAL	Calcarine fissure and surrounding cortex
23	CUN	Cuneus
24	LING	Lingual gyrus
25	SOG	Superior occipital gyrus
26	MOG	Middle occipital gyrus
27	IOG	Inferior occipital gyrus
28	FFG	Fusiform gyrus
29	PoCG	Postcentral gyrus
30	SPG	Superior parietal gyrus
31	IPL	Inferior parietal, but supramarginal and angular gyrus
32	SMG	Supramarginal gyrus
33	ANG	Angular gyrus
34	PCUN	Precuneus
35	PCL	Paracentral lobule
36	CAU	Caudate nucleus
37	PUT	Lenticular nucleus, putamen
38	PAL	Lenticular nucleus, pallidum
39	THA	Thalamus
40	HES	Heschl gyrus
41	STG	Superior temporal gyrus
42	TPOsup	Temporal pole: superior temporal gyrus
43	MTG	Middle temporal gyrus
44	TPOmid	Temporal pole: middle temporal gyrus
45	ITG	Inferior temporal gyrus


*Due to space limitations, the detailed descriptions (including L, left; R, right) could be found at http://neuro.imm.dtu.dk/wiki/Automated_Anatomical_Labeling.*

### Network Analysis

#### Small-World Properties

Network analyses were performed with the GRETNA toolbox 2.0.0 release (GRETNA^1^) (Wang et al., 2015). All global and nodal metrics were defined by Rubinov and Sporns (2010), which including following: small-world coefficient (σ), clustering coefficient (Cp), characteristic path length (Lp), normalized Cp (γ), normalized Lp (λ), global efficiency (Eg), local efficiency (Eloc), nodal efficiency (Ne), betweenness centrality (Bc), and degree centrality (Dc) (the definitions of all network metrics are listed in Table 2). We used the sparsity threshold range of 0.05–0.23 with an interval of 0.01 to discriminate the between-group difference with 1,000 matched random networks. The sparsity threshold was selected based on the minimum threshold as determined by the average degree of all network nodes at each threshold which should be larger than log (*N*) (*N* = 90, *N* means the total number of nodes), and the max threshold as determined by the sigma of all individual networks must be larger than 1.1 to ensure compliance with the small world structure and was in line with previous studies (Tzourio-Mazoyer et al., 2002; Shu et al., 2018).

**Table 2:** The definitions of global and nodal topological properties in the study.

Global network properties	
Small-world coefficient (sigma, σ)	Sigma = lambda/gamma, a real network would be considered small world if γ > 1 and λ ≈ 1, or σ = λ/γ > 1.
Clustering coefficient (Cp)	Cp is the average clustering coefficient over all nodes, which measures by calculating the fraction of the node’s neighbors that are also neighbors of each other.
Characteristic path length (Lp)	Lp is the average distance of the shortest path between every pair of nodes in all nodes, which indicates the efficiency of information transferred on a network.
Normalized Cp (gamma, γ)	γ = C_preal_/C_prand_, C_prand_ is the mean clustering coefficient of 1,000 matched random networks.
Normalized Lp (lambda,λ)	λ = L_preal_/L_prand_, L_prand_ is the mean shortest path length of 1,000 matched random networks
Global efficiency (Eg)	Eg is defined as the mean value of shortest path length between all pairs of nodes in the network. It is a measure of functional integration.
Local efficiency (Eloc)	Eloc is defined as the inverse of the average shortest path connecting all neighbors of a node. It is a measure of functional segregation.
Nodal network properties	
Nodal efficiency (Ne)	Ne is defined as the efficient between a node and all other nodes in the network, which evaluate the capacity of a given node for information communication.
Betweenness centrality (Bc)	Bc is defined as the fraction of shortest paths passing through a node, which evaluate the contribution of a node on the communication for other nodes.
Degree centrality (Dc)	Dc is defined as the number of edges that a node shares with other nodes in the network, which is a measure of node importance in the network.


#### Hub Distribution

Hub nodes were defined with nodal properties (Ne, Dc, Bc) at least one standard deviation (SD) above the mean nodal properties across all regions in each group. Meanwhile, we further compared between-hemisphere differences in three nodal properties (FDR corrected, *P* < 0.05) in the PI and HC groups.

#### PI-Related Subnetwork Analysis

According to the detailed descriptions in (Zalesky et al., 2010) study, network-based statistic (NBS) connectomes were employed to determinate PI-related subnetwork by the NBS toolkit (version 1.2) (NBS^2^). 10,000 non-parametric permutation tests and NBS corrected (*P* < 0.01) were performed to estimate the significance of each component in identifying the connected subnetworks.

### Statistical Analysis

SPSS 16.0 software (SPSS Inc., Chicago, IL, United States) was used to compare demographic and clinical characteristics. We used the Shapiro–Wilk test to test the normality of all data. We evaluated the differences of education time, age, PSQI, ISI, SAS, and SDS scores in PIs and HCs by the Mann–Whitney *U* test. A χ^2^ test was used to compare the qualitative variables of gender. In MATLAB software, 10,000 non-parametric permutation tests (Nichols and Holmes, 2002) were employed to assess between-group differences in global and regional network metrics after adjusted age, gender, and education levels as covariates. Briefly, all differences were randomly divided into two groups, and the same primary threshold (*P* < 0.05) was set to compare by recalculating the mean differences between the two randomized groups (10,000 permutations). For comparisons of global and nodal metrics, Benjamin–Hochberg false discovery rate (FDR) correction (Benjamini and Hochberg, 1995) was performed to address the multiple comparisons at a significance level of 0.05 (*p* < 0.05). After between-group differences of network metrics were identified in the topological properties and nodal metrics, Spearman’s correlation was performed by SPSS 16.0 software to assess the associations of these nodal metrics with clinical scores (SAS, SDS, ISI, PSQI scores and disease duration) in PI patients, removing age, gender, and education levels as covariates. All differences of network properties were statistically analyzed by SPSS 16.0 software, MATLAB 2016 software (Matlab, MathWorks, United States), and GRETNA 2.0.0 release software (Gretna, Beijing Normal University, China).

### Reproducibility Analysis

To evaluate the reliability and reproducibility of the research results, we repeated network analysis with different thresholds (FN ≥ 1, 2, 3, 5, and 10) in AAL-90 templates and a threshold (FN = 1) in AAL-1024 as previous studies (Bai et al., 2012; Chen et al., 2013). This finding can be replicated with different thresholds and parcellation schemes over the sparisity threshold range of 0.05–0.23 with an interval of 0.01.

## Results

### Demographic and Clinical Characteristics

No significant differences were found in age, gender, and education between the PI and HC groups (*P* > 0.05; Table 3). The 44 PI patients (20 males, mean age: 42.4 ± 12.65 years and 24 females, mean age: 39.83 ± 11.04 years) and 46 HCs (17 males, mean age: 38.88 ± 7.26 years and 29 females, mean age: 39.41 ± 9.81 years) showed significant differences in PSQI, ISI, SAS, and SDS scores (*P* < 0.05, Table 3).

**Table 3:** Demographics and clinical characteristics of all participants.

Characteristic	PI participants (*n* = 44)	HC participants (*n* = 46)	*P*-value
Age (years)	40.59 ± 11.51	39.29 ± 9.25	0.458
Sex (male/female)	20/24	17/29	0.233
Duration of education (yeas)	8.12 ± 3.36	7.45 ± 5.21	0.372
PSQI	16.49 ± 3.66	2.28 ± 2.46	<0.001
ISI	20.84 ± 3.67	2.35 ± 2.63	<0.001
SAS	47.43 ± 9.52	5.28 ± 10.43	<0.001
SDS	52.03 ± 9.77	6.25 ± 11.50	<0.001


*—Unless otherwise noted, data are expressed as mean ± SD. PSQI, Pittsburgh Sleep Quality Index; ISI, Insomnia Severity Index; SAS, Self-Rating Anxiety Scale; SDS = Self-Rating Depression Scale; PI, primary insomnia; HC, healthy control.*

### Global Topological Organization of Structural Connectome

**FIGURE 1:**
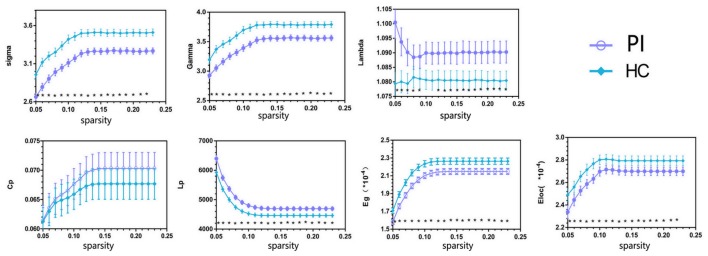
Overall sparsity of group comparison of global network topological properties (Sigma, Gamma, Lambda, Cp, Lp, Eg, and Eloc) of AAL-90 structural connectivity network between the PI and HC groups. The stars indicate the significantly statistical difference between two groups (10,000 permutations, *p* < 0.05, FDR corrected). The vertical bar indicates the standard deviation across subjects. For the abbreviations of network metrics, see Table 2; PI, primary insomnia; HC, healthy control.

Both PIs and HCs showed characteristic small-world topology in the brain structural connectome across all selected thresholds (γ > 1, λ ≈ 1, and σ > 1) compared with 1,000 matched random networks. Compared with HCs, PIs exhibited significantly lower σ (*P* < 0.001), γ (*P* < 0.001), Eg (*P* = 0.005), and Eloc (*P* = 0.035), higher λ (*P* = 0.027) and Lp (*P* = 0.004) (Figure 1).

### Different Hub Regions of Regional Topological Organization of Structural Connectome in Two Groups

**FIGURE 2:**
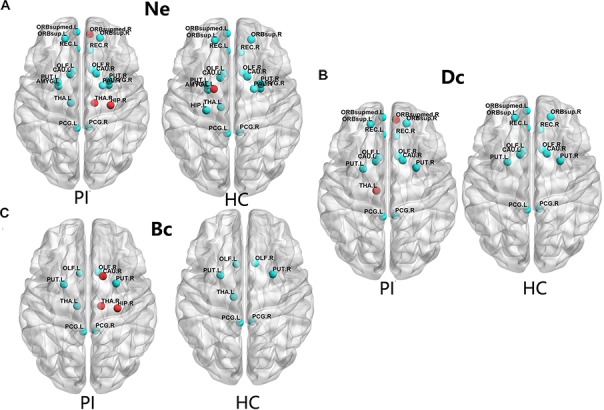
The distribution of the hub regions, left (L) and right (R). The hub regions were identified of three type nodal properties (Ne, Dc, and Bc) at least one standard deviation (SD) above the mean nodal properties across all brain nodes in each group. Nodes with the red color presented unique nodal properties in PI patients compared with HC subjects. Nodes with the cyan point color presented the same hub nodes in both groups. The nodal efficiency was computed in the WM connections with a density of 15%. **(A)** Shows nodal properties in Ne. **(B)** Shows nodal properties in Dc. **(C)** Shows nodal properties in Bc. Ne, nodal efficiency; Bc, betweenness centrality; Dc, degree centrality. For the abbreviations of the brain nodes, see Table 1; L, left; R, right; PI, primary insomnia; HC, healthy control.

**FIGURE 3:**
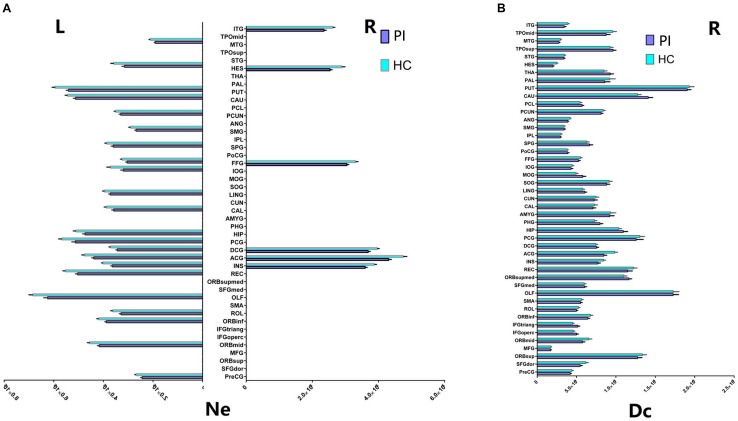
Cortical regions with hemispheric asymmetry in node properties. **(A)** Bars and error bars represent the significant asymmetric effect of Ne (6 right nodes and 22 left nodes) in both hemispheres in structural connectome (*p* < 0.05, FDR corrected). **(B)** Bars and error bars represent significant differences of Dc only in right hemispheres in structural connectome. Ne, nodal efficiency; Dc, degree centrality; L, left; R, right; PI, primary insomnia; HC, healthy control.

The PI patients and HCs had similar hub regions as shown in Figure 2 by cyan color. In nodal efficiency (Ne), PI patients presented twenty regions acting as hubs, while HCs presented nineteen regions (Figure 2, cyan point). Five unique regions of nodal properties (Ne, Dc, and Bc) in PIs were located in ORBsupmed.R, HIP.R, bilateral THA and CAU.R compared with HCs (Figure 2). PAL.L was the only unique Ne region in HC group. More specifically, three different regions were located in the right orbital part of the medial superior frontal gyrus (ORBsupmed.R), HIP.R and right thalamus (THA.R) in PI patients (Figure 2A, red point). The left lenticular nucleus and pallidum (PAL.L) was the only unique Ne region in HC group (Figure 2A, red point). In the degree centrality (Dc), fifteen hub regions were commonly identified in each group. Two unique regions in PI patients were found, locating in the ORBsupmed.R and THA.L (Figure 2B, red point). Meanwhile, PI patients presented three different brain nodes in the betweenness centrality (Bc) compared with HCs, including right caudate nucleus (THA.R), HIP.R and caudate nucleus (CAU.R) (left of Figure 2C, red point). These common hub regions shared by both patients and HCs were often bilateral brain regions, including orbital part of superior frontal gyrus (ORBsup), CAU, olfactory cortex (OLF), rectus gyrus (REC), posterior cingulate gyrus (PCG), and lenticular nucleus and putamen (PUT), which were found in more than two types of hub regions. Above all, five different hub regions were identified between the two groups with four regions in the right brain structural connectome except THA.L. There was a interesting asymmetric effect of the nodal properties (Ne, Dc, and Bc) between the PI and HC groups (*P* < 0.05, 10,000 non-parametric permutation tests) (Figure 3).

### Different Nodal Properties of Both Hemispheres in Structural Connectome

Our study further suggested that the nodal properties showed significant hemispheric effect (Ne, Dc, and Bc) between the PI and HC groups (*P* < 0.05, 10,000 non-parametric permutation tests). All patients with Ne showed lower Ne in PI patients compared with HC group, including all unique hub nodes in PI patients. In comparison with the HC group, Ne in PI patients showed a significant asymmetric effect (6 right nodes and 22 left nodes) in both the hemispheres in structural connectome (*P* < 0.05, Benjamin–Hochberg FDR correction, FDR for short). Specifically, REC.L, INS.L, and PUT.L showed lower Ne in the PI left hemisphere (*P* < 0.001, FDR corrected, Figure 3). Similarly, the Dc of all right brain regions showed significant differences between the two groups (*P* < 0.05, no FDR corrected, Figure 3), including all right unique hub nodes in PI patients. But the Dc of left hemisphere and the Bc of both hemispheres showed no significant differences when compared between PI patients and the HCs. For the abbreviations of the brain nodes, these can see Table 1 of the main manuscript.

### PI-Related Subnetwork Connectivity

**FIGURE 4:**
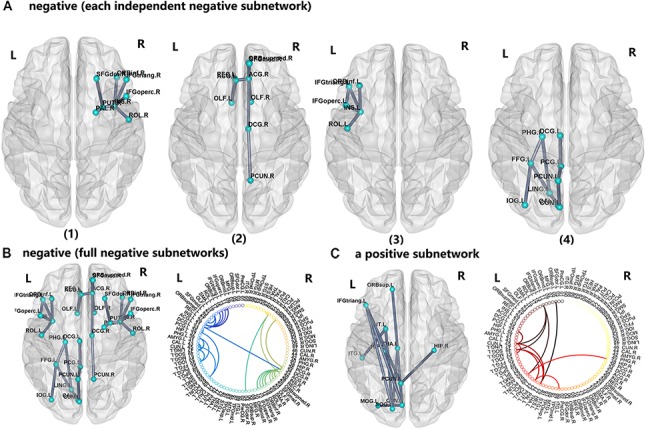
The network-based statistic (NBS) shows the disrupted structural connection in the insomnia participants compared with the HCs. **(A,B)** Four structural subnetworks with decrease connections were separated (*P* < 0.01, NBS corrected). **(C)** One subnetwork with increase connections was uniformly significantly increased (*P* < 0.001, NBS corrected). For the abbreviations of the brain nodes, see Table 1; L, left; R, right.

For the PI group, five subnetworks were revealed in the WM connectome (details listed in Figure 4 and Table 4) that were related to the limbic cortico-basal-ganglia circuit and default-mode networks (prefrontal cortex, CUN.L, left superior occipital gyrus, left middle occipital gyrus and left precuneus). Four decreased structural subnetworks were separated and mainly distributed in the bilateral prefrontal cortex, left occipital and temporal cortex, and many middle line regions of the brain (*P* < 0.01, NBS corrected; Figure 4). Most regions of the uniquely increased subnetworks were located in the left hemisphere, but with right hippocampus (HIP.R, *P* < 0.01, NBS corrected; Figure 4C). Moreover, our study showed the frontal cortex had lower the connection strength with the bilateral INS, ACG, PUT.R and increased the connection strength with THA.L, CUN.L, left precuneus (PCUN.L), and left superior occipital gyrus (SOG.L).

**Table 4:** Regions of four insomnia-related subnetworks.

Index	Region 1 abbreviations	Region 2 abbreviations
**Regions of decrease insomnia-related subnetwork 1**		
(12,30)	IFGoperc.R	INS.R
(14,30)	IFGtriang.R	INS.R
(16,30)	ORBinf.R	INS.R
(18,30)	ROL.R	INS.R
(4,74)	SFGdor.R	PUT.R
(30,74)	INS.R	PUT.R
(74,76)	PUT.R	PAL.R
**Regions of decrease insomnia-related subnetwork 2**		
(21,27)	OLF.L	REC.L
(21,31)	OLF.L	ACG.L
(22,32)	OLF.R	ACG.R
(24,32)	SFGmed.R	ACG.R
(26,32)	ORBsupmed.R	ACG.R
(31,32)	ACG.L	ACG.R
(32,34)	ACG.R	DCG.R
(34,68)	DCG.R	PCUN.R
**Regions of decrease insomnia-related subnetwork 3**		
(11,13)	IFGoperc.L	IFGtriang.L
(11,29)	IFGoperc.L	INS.L
(13,29)	IFGtriang.L	INS.L
(15,29)	ORBinf.L	INS.L
(17,29)	ROL.L	INS.L
**Regions of decrease insomnia-related subnetwork 4**		
(33,35)	DCG.L	PCG.L
(43,45)	CAL.L	CUN.L
(39,47)	PHG.L	LING.L
(43,47)	CAL.L	LING.L
(39,55)	PHG.L	FFG.L
(47,55)	LING.L	FFG.L
(53,55)	IOG.L	FFG.L
(35,67)	PCG.L	PCUN.L
(45,67)	CUN.L	PCUN.L
**Regions of increase insomnia-related subnetwork 5**		
(13,45)	IFGtriang.L	CUN.L
(37,45)	HIP.L	CUN.L
(13,49)	IFGtriang.L	SOG.L
(45,51)	CUN.L	MOG.L
(13,67)	IFGtriang.L	PCUN.L
(38,67)	HIP.R	PCUN.L
(49,73)	SOG.L	PUT.L
(5,77)	ORBsup.L	THA.L
(45,77)	CUN.L	THA.L
(49,77)	SOG.L	THA.L
(73,89)	PUT.L	ITG.L


### Relationship Between Unique Nodal Properties of Hub Regions and Clinical Characteristics in PI Patients

**FIGURE 5:**
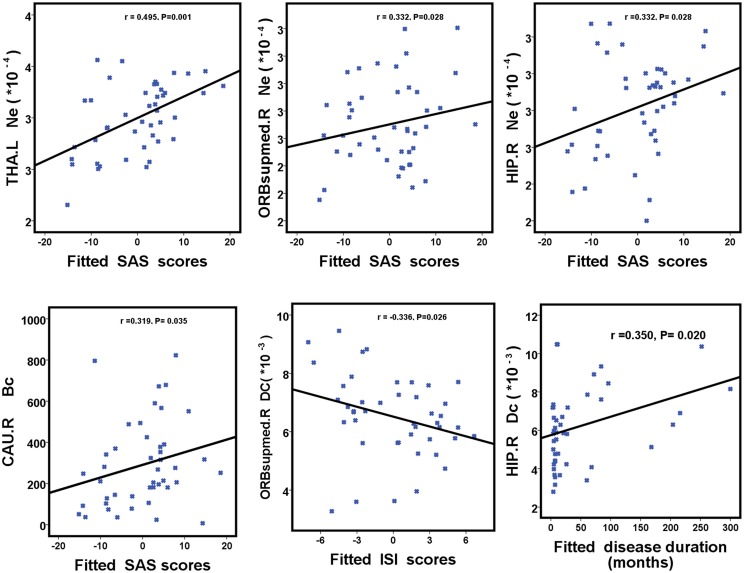
Scatterplots show the relationships between hub nodal properties and clinical characteristics in participants with PI. The fitted values indicate the residuals of PSQI, ISI, SAS, and SDS scores after removing the effects of age, sex, and years of education. PI, primary insomnia; HC, healthy control; SAS, the Self-Rating Anxiety Scale; SDS, Self-Rating Depression Scale; PSQI, Pittsburgh Sleep Quality Index; ISI, Insomnia Severity Index.

We further assessed the associations of hub nodal properties (Ne, Dc, and Bc) with clinical characteristics in PI patients (Figure 5 and Table 5). There were significant positive correlations between SAS scores with unique hub nodal properties in PI patients (Ne of THA.L, *r* = 0.495, *P* = 0.001; Ne of ORBsupmed.R, *r* = 0.332, *P* = 0.028; Ne of HIP.R, *r* = 0.332, *P* = 0.028; Bc of CAU.R, *r* = 0.319, *P* = 0.035). Meanwhile, significant positive correlations between SDS scores and Ne of THA.L (*r* = 0.438, *P* = 0.003) were observed. In addition, a significant negative correlation between Dc of ORBsupmed.R with ISI scores (*r* = -0.336, *P* = 0.026) was observed. Moreover, significant positive correlations between disease duration and unique hub nodal properties of PI patients (Dc of ORBsupmed.R, *r* = 0.308, *P* = 0.042; Dc of HIP.R, *r* = 0.350, *P* = 0.020; Bc of HIP.R, *r* = 0.328, *P* = 0.030) were observed. However, no significant correlation between unique hub nodal values and PSQI scores was found in PI patients.

**Table 5:** Relationship between unique nodal properties of hub regions and clinical characteristics in PI patients.

Brain hub region	Nodal properties	Clinical characteristics	*r*	*P*-value
ORBsupmed.R	Ne	SAS scores	0.332	0.028
	Dc	ISI scores	-0.336	0.026
	Dc	Disease duration	0.308	0.042
HIP.R	Ne	SAS scores	0.332	0.028
	Dc	Disease duration	0.350	0.020
	Bc	Disease duration	0.328	0.030
THA.L	Ne	SAS scores	0.495	0.001
	Ne	SDS scores	0.438	0.003
CAU.R	Bc	SAS scores	0.319	0.035


*PIQS, Pittsburgh Sleep Quality Index; ISI, Insomnia Severity Index; SAS, Self-Rating Anxiety Scale; SDS, Self-Rating Depression Scale; PI, primary insomnia.*

## Discussion

We found that PIs had small-world architectures with lower global and local efficiencies than HCs. Asymmetric changes in unique hub nodes and five disrupted subnetworks of the PI WM network suggested that topological alterations were mostly involved in the limbic cortico-basal-ganglia circuit (Haynes et al., 2018) and default-mode networks (DMN) (Van Calster et al., 2017). Additionally, there were significant correlations between clinical characteristics (SAS, SDS, ISI scores and disease duration) and unique hub nodal properties in PIs.

In our study, anatomical topology analysis revealed that the two study groups had characteristic small-world organization, which was consistent with the finding of previous studies (Lu et al., 2017b; Ma et al., 2018). Eg and Eloc were used as measures of functional integration and segregation, respectively (Rubinov and Sporns, 2010). Our finding implied neuroinformation transfer disruption in PI. A previous study (Crossley et al., 2014) suggested hub nodes may serve as important centers of information integration and segregation and may be targets of priority attack in diseases. Therefore, we speculated that these unique hub nodes may be affected in PI.

Here, abnormal unique hub nodes were indeed observed, including the prefrontal cortex, right HIP, right caudate nucleus (CAU.R) and THA.R in PIs compared with those in HCs. These hub nodes all are part of the limbic cortico-basal-ganglia circuit. Abnormal prefrontal cortexes in PIs have been reported by many previous studies; for example, Li S. et al. (2018) demonstrated that functional connections decreased in the right fronto-parietal network regions, including the superior frontal gyrus, which are linked to working memory and attention. Using EEG, Muzur et al. (2002) demonstrated that prefrontal cortex influences executive functions in PIs. Furthermore, whole-brain VBM studies demonstrated that orbitofrontal gray matter volume (Altena et al., 2010) and density (Stoffers et al., 2012) were associated with PI complaints. de Vivo et al. (2016) used an adolescent mouse model to investigate the ultrastructure of the frontal cortex that can predict and identify the effects of sleep and sleep loss. We found a negative correlation between the Dc of ORBsupmed.R and ISI scores in structural networks among PIs. Our study, as well as these previous studies, suggested that the prefrontal cortex may be associated with abnormal function or structure in PI. HIP is associated with information consolidation and working memory in sleep-related emotional processing (Murkar and De Koninck, 2018). Despite the inconsistent HP volume results of previous studies (Joo et al., 2014; Li M. et al., 2018), recent animal model studies (Lopez-Virgen et al., 2015; de Vivo et al., 2016; Wadhwa et al., 2017) provided compelling evidence that changes in neuronal ultrastructure in HIP are associated with sleep loss or deprivation. We speculated that these inconsistencies in neuroimaging findings with regard to PI are associated with clinical heterogeneity and use of various techniques and designs for the assessment of substructures in HIP. Our results provide further evidence to support the relation between HIP and PI severity, because PIs were susceptible to negative emotions and had consistent consolidation of negative or dreadful information. CAU is associated with executive dysfunction and control of the sleep–wake behavior, which promotes frequent sleep–wake transitions (Qiu et al., 2010; Stoffers et al., 2014). THA can regulate autonomic and endocrine activation and is associated with a hyperarousal state in PI (Lugaresi, 1992). We speculated that abnormalities in the hub nodes of THA and CAU may be associated with these difficulties in maintaining sleep and early morning awakening owing to frequent sleep–wake transitions in PI processing. Meanwhile, in the disrupted subnetworks, we found decreased connections of the frontal cortex with the bilateral INS, ACG, and PUT.R and increased connections of the frontal cortex with THA.L, CUN.L, PCUN.L and left superior occipital gyrus (SOG.L). Our results further showed the presence of complex and neuroinformation interaction in these hub regions. PIs often have a high comorbidity incidence associated with PI and anxiety/depression. Poor PI quality and cognitive emotional hyperarousal predisposition might trigger and maintain a negative cascade (Watling et al., 2017). Therefore, we speculated that these hub nodes may be associated with abnormal emotional reactivity, such as depression and anxiety in PI, which was supported by significant correlations of emotional scores (SAS and SDS) with the nodal properties of the ORBsupmed.R, CAU.R and THA.R in PIs. Our findings demonstrate that changes in the hub topological properties of the limbic cortico-basal-ganglia circuit may be related to the underlying symptoms of PI, especially in sleep-dependent emotional processing.

Meanwhile, some hub regions and PI-related subnetworks were related to DMN. A DMN is an essential network in the human brain and plays important roles in memory, dreaming, auditory/visual processing, self-awareness and self-processing operations (Domhoff and Fox, 2015; Zuo et al., 2016). Malfunctioning of the DMN may lead to sustained sleep difficulties and sleep architecture disturbances in PIs, particularly, decrease in connectivity between the DMN and HIP can increase sleep depth (Regen et al., 2016). Our NBS results revealed that PIs exhibited increased connectivity in the left hemisphere between many regions in the DMN with right hippocampus, which further demonstrated that the DMN plays an important role in PI processing.

Additionally, we interestingly found asymmetric distribution of these abnormal hub nodes in PIs, which may be due to have several reasons. First, the result might be associated with anatomical and functional lateralization in the two hemispheres. The left hemisphere of right-handed individuals plays a leading role in language, auditory and visual processing (Li et al., 2014; Shu et al., 2015), while the right hemisphere plays an important role in spatial attention, emotion and memory (Li et al., 2014). Over the last 3 years, however, studies have shown that the abnormal asymmetric topological properties of both hemispheres are associated with neurophysiological mechanisms in some diseases, such as AD (Yang et al., 2017), schizophrenia (Sun et al., 2017), and autism spectrum disorder (Sun et al., 2017). PIs often have high reactivity with regard to the functions of emotion and memory, which are closely related to the right hemisphere. Our findings showed that most abnormal hub nodes were located in the right limbic cortico-basal -ganglia circuit and were related to patient symptoms (clinical scores and disease duration). PIs also have heightened sensitivity, dreaminess and self-awareness (Domhoff and Fox, 2015; Zuo et al., 2016), and this is closely associated with the left hemisphere. This study also showed increased connections of DMN in the left hemisphere on NBS analysis. Therefore, we speculated that the asymmetric topology in structural networks provides novel insights into the neural substrates underlying patient symptoms, which requires further investigation. Second, asymmetric changes in nodal properties and disrupted subnetworks in both hemispheres might lead to the asymmetric distribution of these unique hub nodes. The observed rightward asymmetric hub nodes were attributed to changes in the complex topological properties of the left hemisphere in PIs, which is consistent with the findings in a previous AD study (Yang et al., 2017). Third, the definition approach for the WM network had a great effect on the constructed networks and results. Li S. et al. (2018) performed DTI and suggested disruption in the integrity of the right lateralized WM in PIs.

### Limitations

The present study has some limitations. First, cognitive functions were not evaluated in this study, which limited the evaluation of the potential impact of cognitive function in PIs. Second, the sample size of PIs was relatively small. Third, the study was limited by the hardware used. We constructed WM networks according to DTI data using the deterministic fiber-tracking algorithm, which has been often used for DTI data with ‘not-so-good’ quality. However, deterministic tractography has some deficiencies in estimating the crossing fibers. Further investigations involving probabilistic fiber-tracking algorithms and 3T MR scanners are required.

## Conclusion

Using DTI and graph theory analysis, we demonstrated abnormal hub nodal properties and subnetworks involving the limbic cortico-basal-ganglia circuit and DMN in PIs. Moreover, the altered network architecture may be related to the neural substrates underlying patient symptoms and the neurophysiologic mechanisms involved in PI.

## Author Contributions

YW and GJ conceived and designed the experiments. YW, ML, GX, GL, YY, SF, and KH acquired the data. SZ, XM, JY, ClL, GX, CoL, and TW performed the clinical data, which YW, CgL, and ML analyzed. YW and GJ wrote the article, which all authors reviewed and approved for submission.

## Conflict of Interest Statement

The authors declare that the research was conducted in the absence of any commercial or financial relationships that could be construed as a potential conflict of interest.

## Footnotes

1 http://www.nitrc.org/projects/gretna

2 https://www.nitrc.org/projects/nbs

## Abbreviations

DTI: diffusion tensor imaging
FA: fractional anisotropy
FN: streamline number
HC: healthy control
HCs: healthy control subjects
PI: primary insomnia
PIs: primary insomnia patients
WM: white matter
